# The Evolutionary Advantage in Mammals of the Complementary Monoallelic Expression Mechanism of Genomic Imprinting and Its Emergence From a Defense Against the Insertion Into the Host Genome

**DOI:** 10.3389/fgene.2022.832983

**Published:** 2022-03-03

**Authors:** Tomoko Kaneko-Ishino, Fumitoshi Ishino

**Affiliations:** ^1^ School of Medicine, Tokai University, Isehara, Japan; ^2^ Research Institute, Tokyo Medical and Dental University, Tokyo, Japan

**Keywords:** complementation, genome innovation, evolutionary trade-off, insertion of exogenous DNA, host defense hypothesis

## Abstract

In viviparous mammals, genomic imprinting regulates parent-of-origin-specific monoallelic expression of paternally and maternally expressed imprinted genes (*PEGs* and *MEGs*) in a region-specific manner. It plays an essential role in mammalian development: aberrant imprinting regulation causes a variety of developmental defects, including fetal, neonatal, and postnatal lethality as well as growth abnormalities. Mechanistically, *PEGs* and *MEGs* are reciprocally regulated by DNA methylation of germ-line differentially methylated regions (gDMRs), thereby exhibiting eliciting complementary expression from parental genomes. The fact that most gDMR sequences are derived from insertion events provides strong support for the claim that genomic imprinting emerged as a host defense mechanism against the insertion in the genome. Recent studies on the molecular mechanisms concerning how the DNA methylation marks on the gDMRs are established in gametes and maintained in the pre- and postimplantation periods have further revealed the close relationship between genomic imprinting and invading DNA, such as retroviruses and LTR retrotransposons. In the presence of gDMRs, the monoallelic expression of *PEGs* and *MEGs* confers an apparent advantage by the functional compensation that takes place between the two parental genomes. Thus, it is likely that genomic imprinting is a consequence of an evolutionary trade-off for improved survival. In addition, novel genes were introduced into the mammalian genome via this same surprising and complex process as imprinted genes, such as the genes acquired from retroviruses as well as those that were duplicated by retropositioning. Importantly, these genes play essential/important roles in the current eutherian developmental system, such as that in the placenta and/or brain. Thus, genomic imprinting has played a critically important role in the evolutionary emergence of mammals, not only by providing a means to escape from the adverse effects of invading DNA with sequences corresponding to the gDMRs, but also by the acquisition of novel functions in development, growth and behavior via the mechanism of complementary monoallelic expression.

## Introduction

Genomic imprinting is widely distributed in the viviparous mammals, the therians, comprising marsupials and eutherians (Reik and Walter 2001; Renfree et al., 2009; Barlow and Bartolomei 2014), yet it is an unusual biological mechanism in that it seems to runs counter to two main pillars of modern biology: it is an apparent exception to the rule of Mendelian genetics that presupposes biallelic expression from two parental alleles, and it is inconsistent with the Darwinian theory of evolution at first glance due to the apparent disadvantage (Mann and Lovell-Badge, 1984; McGrath and Solter 1984; Surani et al., 1984; Cattanach and Kirk 1985) of the monoallelic expression of certain essential/important genes in development. The evolutionary advantage it nevertheless confers as the result of a partial, functional haploidy despite such defects has long been debated. Perhaps the most widely accepted account is the conflict/kinship hypothesis (Moore and Haig, 1991; Wilkins and Haig 2003; Haig 2004), which proposes that genomic imprinting arose as a consequence of a conflict of interest between maternally and paternally derived genomes, driven by a need for prenatal resource control: *PEGs* promote embryonic growth while *MEGs* repress it. Substantial numbers of imprinted genes fit this hypothetical scenario, so it has become generally accepted and its topic covered by many reviews (Reik and Walter, 2001; Edwards et al., 2019; Hanna and Kelsey 2021). In this review, we first introduce the unique nature of genomic imprinting regulated by gDMRs, then, revisit another host defense hypothesis that proposes that genomic imprinting arose as a consequence of the DNA methylation machinery as a defense against repetitive/foreign elements (Barlow 1993). We do this from the viewpoint of the origin of gDMRs (Suzuki et al., 2011; Renfree et al., 2013; Kaneko-Ishino and Ishino 2015, 2019) and the current knowledge on the molecular mechanisms underlying the establishment of the gDMRs in germ cells (Chotalia et al., 2009; Veselovska et al., 2015; Hanna and Kelsey 2021) as well as their protection from a global DNA demethylation wave that occurs in preimplantation embryos (Li et al., 2008; Smallwood et al., 2011; Kobayashi et al., 2012; Takahashi et al., 2019). Finally, we reexamine the advantage conferred by complementary monoallelic expression (Kaneko-Ishino et al., 2003, 2006), such as functional compensation (Renfree et al., 2013; Kaneko-Ishino and Ishino 2015, 2019) and the innovation of genomic function (Kaneko-Ishino and Ishino, 2012). Recently, non-canonical genomic imprinting regulated by histone 3 lysine 27 trimethylation (H3K27me3) has been reported in the placenta in a lineage-specific manner (Okae et al., 2014; Inoue et al., 2017a, 2017b, 2018; Hanna et al., 2019; Inoue et al., 2020; Hanna and Kelsey 2021), but we shall mainly focus on the canonical genomic imprinting regulated by gDMRs that is commonly conserved in therian and eutherian mammals.

## Uniqueness of Mammalian Genomic Imprinting Among Vertebrates

Pronuclear transplantation experiments demonstrated that both parthenogenetic and androgenetic embryos which exclusively possess maternally and paternally-derived genomes, respectively, cannot develop to term, and exhibit early embryonic lethality: the former exhibited severe placental defects, while the latter had severe embryonic growth retardation (Mann and Lovell-Badge, 1984; McGrath and Solter 1984; Surani et al., 1984)*.* The complete absence of parthenogenesis is a unique feature of viviparous mammals because parthenogenesis is often observed both naturally and experimentally in vertebrates such as birds, reptiles, amphibians, and fish (Ramachandran and McDaniel 2018; Fujita et al., 2020). The differences between the paternally and maternally-derived genome have also been shown by genetic experiments using mice with partial paternal or maternal uniparental disomy (Cattanach and Kirk, 1985; Cattanach and Beechey, 1990; Beechey and Evans, 2004). The offspring exhibit lethality at various stages in pre- and postnatal development as well as growth and behavioral abnormalities, leading to the concept of chromosomal imprinted regions. Such genomic imprinting is well accounted for by the presence of paternally and maternally expressed imprinted genes (*PEGs* and *MEGs*). The early embryonic lethality of the parthenogenetic and androgenetic embryos is due to a complete lack of expression of *PEGs* and *MEGs*, respectively. The abnormal phenotypes in the partial uniparental disomy mice are due to the irregular expression of certain *PEGs* and *MEGs* in the imprinted regions in question. From these results, it is clear that several different *PEGs* and *MEGs* play essential/important roles in the current developmental system in the therian mammals, so even their monoallelic expression is advantageous compared with a complete absence of expression, leading to their widespread conservation.

## The Intrinsic Nature of Reciprocal Expression by *PEGs* and *MEGs*

**FIGURE 1:**
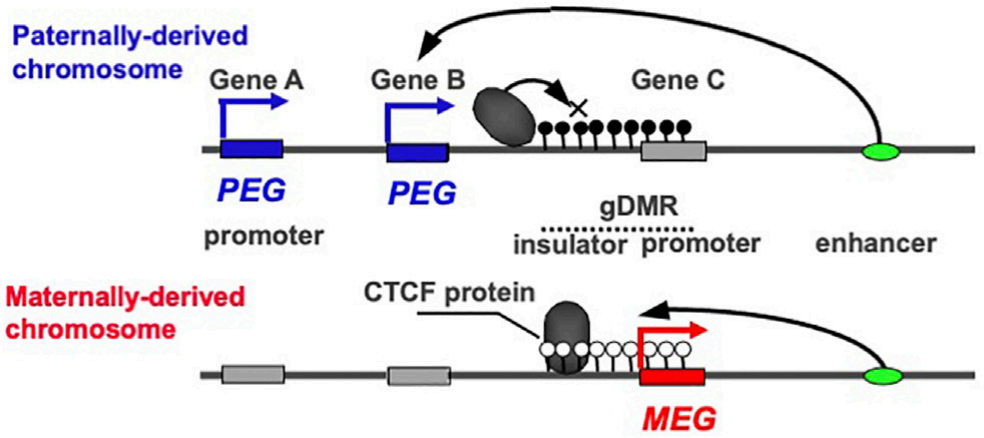
The reciprocal ON-OFF switch of *PEG* and *MEG*. In imprinted regions, *PEG* and *MEG* cannot be co-expressed in *cis*. In the insulator model, they are reciprocally regulated by the DNA methylation status of the gDMR including the insulator sequence. An insulator binding protein, such as CTCF, interrupts the downstream enhancer activity, leading to a repression of Genes A and B without any effect on Gene C. In contrast, DNA methylation on the insulator sequence inhibits the binding of the CTCF protein, leading to the induction of the Genes A and B concurrently with repression of the Gene C via DNA methylation on its promoter contiguous to the insulator sequence. The blue and red boxesindicate paternally and maternally active alleles, respectively, while the gray boxes indicate repressed alleles. Then, the blue and red arrows indicate *PEG* and *MEG* expression, respectively. The white and black circles indicate non-methylated and methylated CpGs, repressively. This is an updated version of Figure 2 in the previous review (Kaneko-Ishino and Ishino 2019).

*PEG* and *MEG* cannot be co-expressed in the *cis* configuration because their expression is under the control of the gDMRs, which are also referred to as imprinting control centers (ICRs), that contain *cis*-regulatory elements (Figure 1). This is a critical part of the intrinsic character of the imprinted regions. In most of the canonical imprinted regions, such *cis*-regulatory elements have long-range effects on the expression of neighboring genes, thus regulating a number of *PEGs* and *MEGs*. In the insulator model, the insulator sequences are utilized so the *cis*-regulatory elements are able to inhibit downstream enhancer activity (Bell and Felsenfeld 2000; Hark et al., 2000) (Figure 1). In the antisense models, the promoters of antisense transcripts interfere with the transcription of downstream genes (Stoger et al., 1993; Wutz et al., 1997). In certain cases, two separate regulatory regions are used to control the entire imprinted region. In the bipartite model, two regulatory units are required for the activity of a bipartite imprinting center, one containing the gDMR while the other may act with the former in gametes in the *cis* configuration, although the precise molecular mechanism remains unknown (Nicholls and Knepper 2001; Wu et al., 2012). In other cases, both the gDMRs and secondary DMRs (sDMRs) that are established in a manner depending on the former after fertilization, play essential roles (Takada et al., 2000, 2002; Lopes et al., 2003; Kagami et al., 2008; Kagami et al., 2015; Takahashi et al., 2009; Zhou et al., 2010; Beygo et al., 2015). Regardless of which molecular mechanism model applies**,** DNA methylation of either one of the parental gDMRs inhibits the activity of the *cis*-regulatory elements, thereby undoing the off state and leading to the complementary expression of *PEG* and *MEG* (Kaneko-Ishino et al., 2003; Kaneko-Ishino et al., 2006)*.*

**FIGURE 2:**
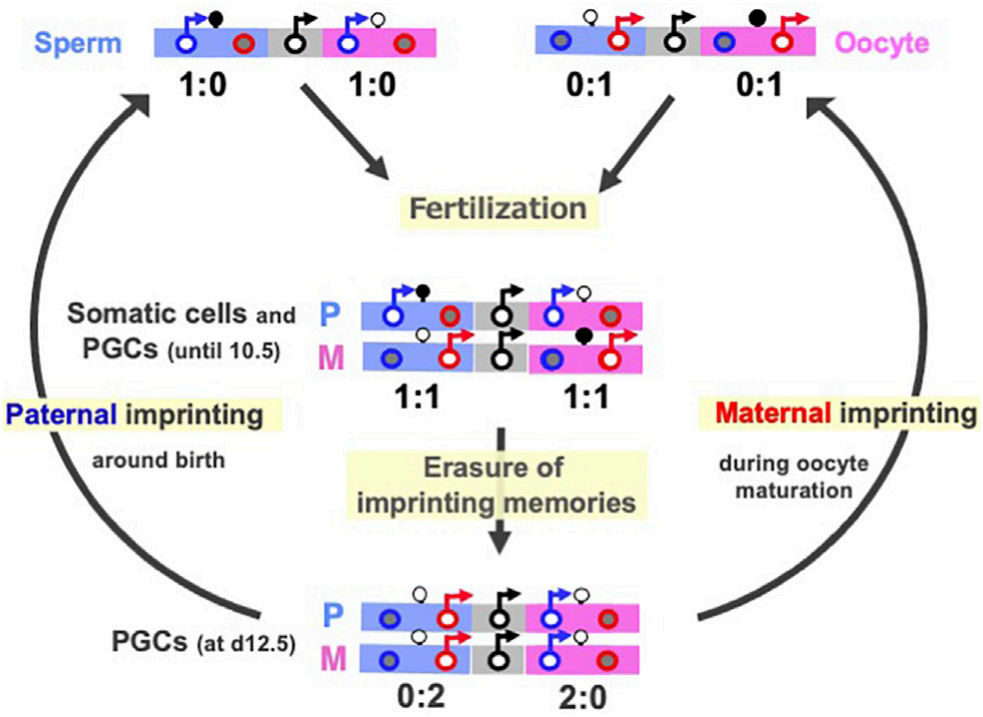
Cycle of genomic imprinting memory. Top: Sperm (left) and oocytes (right) have imprinted memories to express only *PEGs* and *MEGs* in somatic cells. Their expression patterns here represent those of androgenetic and parthenogenetic embryos, respectively. Second: The expression profiles of the imprinted genes in paternal and maternal imprinted regions in somatic cells and PGCs until at most day 10.5 (Miki et al., 2005; Yamazaki et al., 2005) This is reestablished by a combination of the sperm and oocyte patterns. Bottom: The expression profiles of imprinted genes in the default states of genomic imprinting (i.e., without any DNA methylation), such as day 12.5 PGC cloned embryos. The black, blue and red circles represent normal biallelic, paternally and maternally expressed genes, respectively. There are two types of imprinted regions, paternally (blue) and maternally imprinted (pink) reginos, in which gDMRs are methylated paternally and maternally, respectively. The erasure of imprinted memories in PGCs occurs around d10.5 and completed by d12.5, then gDMR methylation in the paternally imprinted regions (Paternal imprinting) occurs during prospermatogonia development around the time of birth, while that in the maternally imprinted regions (Maternal imprinting) occurs during oocyte maturation (arrows from the bottom to top). *PEG* and *MEG* cannot be co-expressed in *cis* in any stages of this cycle.

DNA methylation of the gDMRs, referred to as imprinted memories, are established by DNA methyltransferase 3A (DNMT3A) and its catalytically inactive cofactor DNMT3L in either oocytes or spermatogonia (Okano et al., 1999; Bourc’his et al., 2001; Hata et al., 2002; Bourc’his and Bestor 2004; Kaneda et al., 2004), thus leading to the establishment of maternal and paternal gDMRs in the oocytes and sperm (Figure 2A). Such gDMRs are maintained in the somatic cells throughout life (Figure 2, middle), while in germ cells, it is completely erased for individuals in the next generation (Figure 2, bottom). From embryonic day 10.5 (d10.5), the erasure of genomic imprinting memory occurs in primordial germ cells (PGCs) through the passive replication-dependent DNA demethylation mechanism (Hackett et al., 2013; Yamaguchi et al., 2013a, 2013b; Kagiwada et al., 2013) and possibly together with the active mechanism catalyzed by ten-eleven translocation (*TET*) methylcytosine dioxygenases (Kawasaki et al., 2014). Importantly, clone embryos produced from d12.5 PGCs in the default mode of genomic imprinting (no DNA methylation of parental gDMRs) (Hajkova et al., 2002; Lee et al., 2002; Szabo et al., 2002) also exhibit early embryonic lethality (Lee et al., 2002; Yamazaki et al., 2005) similar to the parthenogenetic and androgenetic embryos. They express only *MEGs* in the paternally imprinted regions and only *PEGs* in the maternally imprinted regions, that is, the *PEGs* in the former and *MEGs* in the latter are repressed, respectively (Figure 2, middle). These results indicate that DNA methylation on the gDMRs is necessary to induce the repressed genes in the default state, indicating the strict requirement of having both paternal and maternal epigenotypes, leading to the complementary expression of *PEGs* and *MEGs* (Kaneko-Ishino et al., 2003; Kaneko-Ishino et al., 2006).

Paternal and maternal imprinting memories are then reestablished depending on the individual’s sex by DNA methylation on the paternal and maternal gDMRs in the paternal and maternal imprinted regions, respectively (Davis et al., 1999; Li et al., 2004; Lucifero et al., 2004; Hiura et al., 2006) (Figure 2, arrows from bottom to top), and as a result, the “imprinted” patterns of sperm and oocytes are completed (Figure 2, top). All of these changes in the expression pattern are consistent with the reciprocal regulation of *PEGs* and *MEGs* in a manner that depends on the DNA methylation status of the gDMRs (Figure 1). Taken together, DNA methylation at the gDMRs can be considered to serve as a major epigenetic mark in the life cycle of genomic imprinting and has recently been referred to as “canonical imprinting” in contrast to histone modification-dependent imprinting, as described later.

## The Origin of gDMRs

**FIGURE 3:**
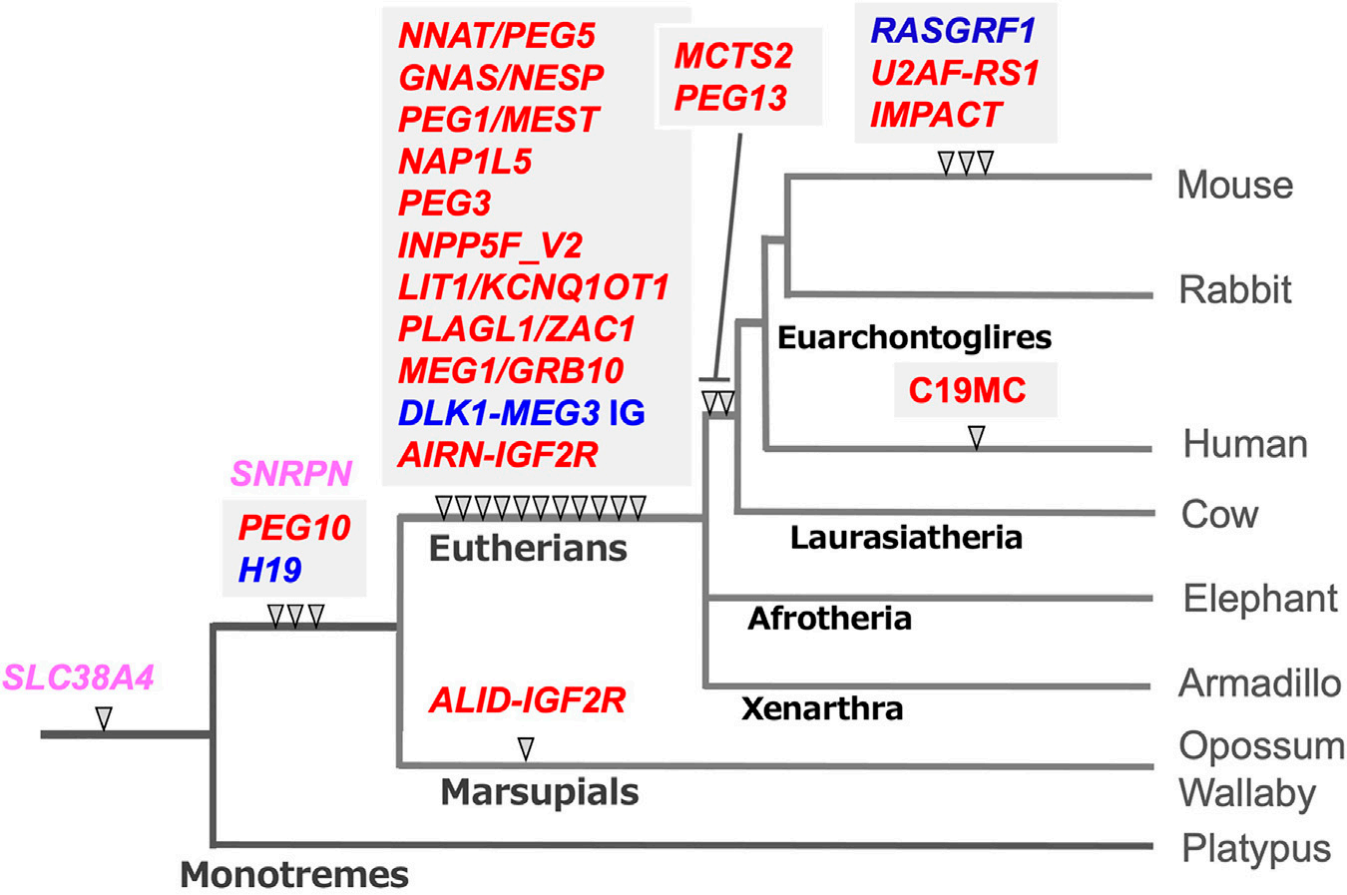
The emergence of gDMR sequences coincides with the onset of imprinted regulation in mammals. In most cases, the emergence of gDMR sequences, the DNA sequences corresponding to the gDMRs, correlate well with the establishment of imprinted regions in mammalian evolution. The arrowheads indicate when each gDMR sequence appeared in the mammalian lineage tree. Blue and red represent that imprinted regulation started as the paternally and maternally imprinted regions, respectively. Pink represents the maternally imprinted regions in which the emergence of DMR sequences preceded the onset of imprinted regulation, for example, *SLC38A4*-and *SNRPN*-DMRs (see the text for the details). It should be noted that mouse *Slc38a4* has recently been recognized as an imprinted gene regulated by both canonical and non-canonical imprinting mechanisms (Okae et al., 2014; Inoue et al., 2017a; Bogutz et al., 2019). This is an updated version of Figure 6 in our previous review (Kaneko-Ishino and Ishino 2019).

Genomic imprinting is observed in eutherian and marsupial mammals, but the number of imprinted genes in eutherians is much larger than in marsupials (Cattanach and Beechey 1990; Killian et al., 2000; O’Neill et al., 2000; Beechey and Evans, 2004; Suzuki et al., 2005; Suzuki et al., 2007). In addition, there exist several lineage- and species-specific imprinted genes (Hayashizaki et al., 1994; Plass et al., 1996; Hagiwara et al., 1997; Smith et al., 2003; Wood et al., 2007; Das et al., 2012). In her host defense hypothesis, Denise Barlow implied that the origin of genomic imprinting lies in an existing biochemical system, such as DNA methylation, that serves to neutralize foreign invading DNA, such as retroviruses, direct repeats, and retrotransposons (Barlow et al., 1991; Neumann and Barlow. 1995). We had a similar idea and independently sought imprinted genes of exogenous origin by comprehensive screening of *PEGs* and *MEGs* (Kaneko-Ishino et al., 1995; Kuroiwa et al., 1996; Miyoshi et al., 1998; Miyoshi et al., 2000; Ono et al., 2001). Identification of paternally expressed 10 (*PEG10*), a gene acquired from a retrovirus (Ono et al., 2001; Ono et al., 2006), led to the finding that the *PEG10*-gDMR in its promoter region emerged with *PEG10* itself in a common ancestor of therian mammals (Suzuki et al., 2007). Subsequent comprehensive comparative genome analysis has revealed that the DNA sequences corresponding to the gDMR in the canonical imprinted regions are derived from inserted DNA sequences (Suzuki et al., 2011; Renfree et al., 2013; Kaneko-Ishino and Ishino 2019). In most cases, such insertion events correlate well with the time when their imprinting regulation activity started as paternally and maternally imprinted regions (Figure 3, blue and red, respectively), providing strong support for the host defense hypothesis. From this perspective, the gDMRs, and most especially the *cis*-functional elements within them, should be recognized as the targets of the genomic imprinting mechanism rather than retroviruses, direct repeats and retrotransposons, as originally proposed (Barlow 1993; Neumann and Barlow 1995).

For example, the *PEG10*-and *H19*-gDMR sequences emerged next to the sarcoglycan epsilon (*SGCE*) promotor and downstream of the Insulin-like growth factor 2 (*IGF2*) gene in both the marsupial and eutherian genomes, respectively (Suzuki et al., 2007; Smits et al., 2008; Kaneko-Ishino and Ishino 2010). Thus, it is certain that their insertion occurred in a common therian ancestor. They were associated with the insertion of the retrovirus-derived *PEG10* and non-coding *H19,* respectively (Table 1). In addition, imprinted paternal expression of *PEG10* (Ono et al., 2001; Suzuki et al., 2007) and reciprocal maternal/paternal expression of *H19/IGF2* (DeChiara et al., 1990, 1991; Bartolomei et al., 1991; O’Neill et al., 2000; Killian et al., 2001; Weidman et al., 2004; Edwards et al., 2008) have been confirmed in both groups. Interestingly, only the *PEG10* promoter is differentially methylated in marsupials. In contrast, the *PEG10*-gDMR extends to the *SGCE* promoter and controls a large imprinted region, including several *MEG*s, in eutherians (Ono et al., 2003; Suzuki et al., 2007).

**TABLE 1:** Newly acquired genes in the imprinted regions. The essential/important imprinted genes that have emerged in therian- and eutherian-specific imprinted regions are summarized.

Orthologs and paralogs	Imprinted gene	Conservation	Origin	Phenotypes in mutant mice: Human diseases
	*PEG10*	therians	Retroviral GAG and POL	Placenta formation, maintenance of fetal capillary network: Llung, liver, panreus cancers, Angelman syndrome?
	*RTL1/PEG11*	eutherians	Retroviral GAG and POL	Placenta, muscle and CNS defects in mice: Kagami-Ogata and Temple syndromes
Acquired genes from retrovirus and genes of unknown origin	*AntiRTL1/AntiPEG11*	eutherians	Unknown	Placenta, muscle and CNS defects in mice by RTL1/PEG11 mRNA regulationl via RNAi: Kagami-Ogata syndrome
	*PEG3*	eutherians	C2H2-type zinc finger protein fused by retrovial GAG	Fetal growth, materal behavior, sex-biased birth rate, thermoregulation: Glioma
	*NNAT/PEG5*	eutherians	Unknown lipoprotein	Cerebellar folding, postnatal growth restriction and adult obesity: Lafora disease, diabetes and cancer
	*NDN*	eutherians	One of *MAGE* family members	Fetal growth defect and partial neonetal lethality: Prader-Willi syndrome
Duplicated genes by retropositioning	*MAGEL2*	eutherians	One of *MAGE* family members	Postnatal growth defect and obsity: Prader-Willi syndrome, Schaaf-Yang syndrome
	*MKRN3*	eutherians	*MKRN1 or MKRN2*	Precosious puberty: Precosious puberty

The *PEG10* and *H19/IGF2* imprinted regions are the first two imprinted regions in mammalian history, while most of the gDMRs emerged in the eutherian genome and constitute eutherian-specific imprinted regions. Subsequently, some lineage-specific imprinted regions appeared, indicating that genomic imprinting arose at many different time points during mammalian evolution and is still continuing to evolve (Suzuki et al., 2011; Das et al., 2012; Renfree et al., 2013). The large miRNA cluster, C19MC, in the human genome is an example of a lineage-specific imprinted region that has emerged in primates (Noguer-Dance et al., 2010; Malnou et al., 2018), implying the existence of other primate- and/or human-specific imprinted regions in the human genome.

Interestingly, the IGF2 receptor (*IGF2R*) is imprinted in both eutherians and marsupials (Killian et al., 2000; Szabo et al., 2000), however, it is regulated by different gDMRs. It is located on intron 2 in the former and intron 12 in the latter (Stöger et al., 1993; Suzuki et al., 2018). Both gDMRs function as promoters of antisense non-coding RNAs, a long Antisense of IGF2R non-protein coding RNA (AIRN) in the former (Stoger et al., 1993) and a short non-coding RNA, Antisense LncRNA in the IGF2R gDMR (ALID), in the latter. It is thus likely that they emerged independently and are regulated by different mechanisms. Marsupial-specific expansion of intron 12, which has occurred as the results of accumulation of a number of transposons, may have contributed to the establishment of the *ALID/IGF2R*-gDMR (Suzuki et al., 2018).

Two apparent exceptions are the DNA sequence corresponding to the Small nuclear ribonucleoprotein polypeptide N (*SNRPN*)*-* and the Solute carrier family 38, member 4 (*SLC38A4*)*-*gDMRs (Figure 3, pink) because their insertion did not coincide with the onset of maternal imprinted regulation. The former regulates the Prader-Willi/Angelman (PWS/AS) region as the PWS-shortest region of deletion overlap (SRO) together with the AS-SRO located 880 bp at a site 35 kb upstream of the *SNRPN* upstream open reading frame (*SNURF*)*-SNRPN* (Nicholls et al., 1998). The DNA sequence corresponding to the *SNRPN-*DMR is conserved in both marsupials and eutherians, although, in marsupials, the *SNRPN,* and Ubiquitin Protein Ligase E3A (*UBE3A*) regions exist on separate chromosomes. The PWS/AS region arose by chromosome rearrangement in eutherians, and the *SNRPN-*DMR located in *SNURF* was established in the newly reconstructed DNA sequence (Rapkins et al., 2006). Similarly, the corresponding sequence to the *SLC38A4-*gDMR already existed in monotremes (Suzuki et al., 2011), but it became differentially methylated only in the rodent lineage in association with the rodent-specific insertion of MT2A retrovirus (Bogutz et al., 2019; Hanna et al., 2019) (see next section). *Slc38a4* has also been recognized as a member of non-canonical imprinted genes in mice, implying that an evolutionary route to the *SLC38A4* imprinted region may be complicated (Okae et al., 2014; Inoue et al., 2017a).

## Molecular Mechanisms Underlying the Establishment of Differential DNA Methylation Patterns in the Female and Male Gametes

So what, then, is the actual biochemical system that serves to neutralize foreign DNA that had been hypothesized by Barlow? Recent work has shown that the gDMRs are established as a consequence of differential responses to the invading DNAs in the oocytes and prospermatogonia as well as to afford subsequent protection from DNA demethylation in preimplantation development (see the next section).

**FIGURE 4:**
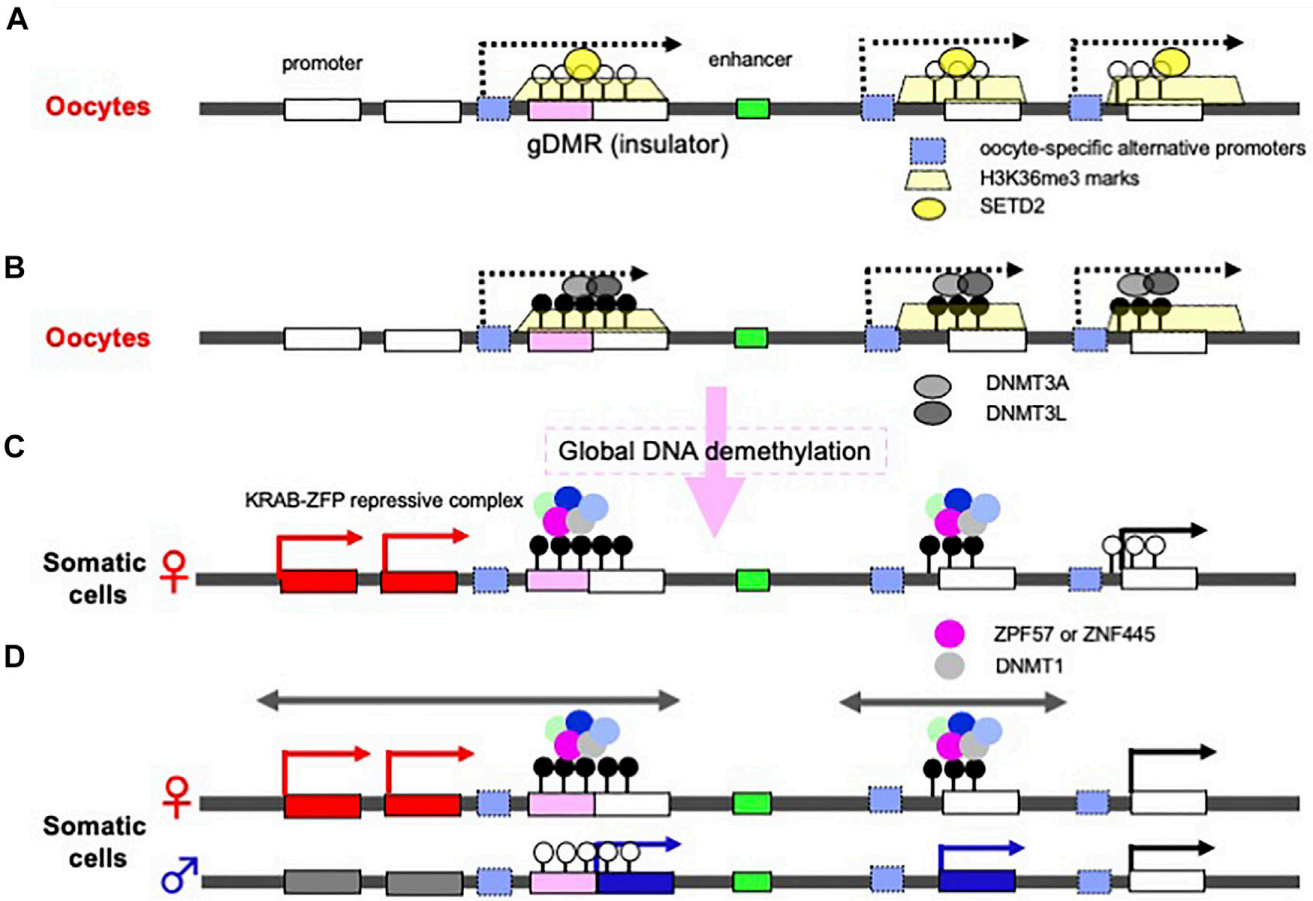
Molecular mechanisms underlying how gDMRs are established and maintained. **(A)** Expression of oocyte-specific transcripts from alternative promoters including LTRs. Oocyte-specific alternative upstream transcripts (dashed arrows) run through the gDMR (a pink box) within gene bodies with deposition of H3K36me3 (yellow trapezoids). In some cases, LTRs are used as the promoters for such transcripts (light blue boxes). **(B)** DNA methylation in gene body regions including maternal gDMRs. The H3K36me3 epigenetic mark guides DNA methylation in the oocytes, leading to the gene body DNA methylation. There are many more differential methylated CpG sequences than gDMRs in oocytes. **(C)** Protection of gDMR DNA methylation from a global DNA demethylation wave. Most of the differentially methylated CpG sequences disappear during preimplantation development due to a global DNA demethylation wave (right), however, both maternal and paternal gDMRs remain protected by a large complex including either ZFP57 or ZNF445, members of the KRAB-ZFPs playing an essential role in repressing invaded retroviruses (left and center). **(D)** The resulting canonical imprinted regions. Double-headed arrows indicate a therian-/eutherian-specific (left) and a species-specific (center) imprinted region in somatic cells. The gDMR DNA methylation is maintained during postimplantation period by symmetric CpG methylation catalyzed by DNMT1 included in the KRAB-ZFPs complex. The differential recognition mechanisms in the paternal and maternal germ cells lead to the imprinted region in somatic cells.

Discovery of the oocyte-specific transcripts spanning the gDMRs has provided an important clue to the mechanism of the establishing of the maternal gDMRs (Chotalia et al., 2009; Veselovska et al., 2015; Smallwood et al., 2011; Hanna and Kelsey 2021). In oocytes, DNA methylation is exclusively on active gene body regions, including the maternally imprinted gDMRs (Kobayashi et al., 2012). Initially, histone lysine methylase SETD2 deposits H3K36me3 (Figure 4A), and subsequently this epigenetic mark guides DNA methylation because it promotes the binding of DNMT3A in the oocyte (Chotalia et al., 2009; Veselovska et al., 2015; Xu et al., 2019; Shirane et al., 2020) (Figure 4B). Interestingly, long terminal repeats (LTRs) of some endogenous viruses (ERVs) are used as the promoters of the oocyte-specific alternative upstream transcripts in the species-specific canonical imprinting (Brind’Amour et al., 2018; Bogutz et al., 2019). These data indicate that the oocyte-specific transcripts from the upstream promoters, such as integrated LTRs, play a critical and fundamental role in the mechanism underlying the establishment of the maternal gDMRs.

On the other hand, in the male germline (prospermatogonia), the lysine methyltransferase NSD1 deposits H3K36me2 in the euchromatic regions included on the paternally imprinted gDMRs, and subsequently this epigenetic mark guides DNA methylation (Shirane et al., 2020). Thus, there is a sexually dimorphic pattern of DNA methylation in mature mouse gametes via the deposition of distinct H3K36 methylation marks (Xu et al., 2019; Shirane et al., 2020). Among the three paternal gDMRs, Ras protein specific guanine nucleotide releasing factor 1 (*Rasgrf1*)-gDMR is known to use an LTR from the RMER4 retrotransposon as an upstream promoter to generate small RNAs that recruit the PIWI-piRNA mechanism to establish *de novo* DNA methylation in spermatogenesis (Watanabe et al., 2011), although the molecular mechanism remains elusive in the other two gDMRs, the *H19*- and *DLK1-MEG3* intergenic (IG)-gDMRs. These data demonstrate that integrated retroviruses (ERVs) and LTR retrotransposons are ingeniously integrated in the DNA methylation of gDMRs that occurs in germ cells.

## Molecular Mechanisms Underlying gDMR Maintenance in pre- and Postimplantation Development

Another important fact is that there are more than 1,600 differentially methylated CpG islands in oocytes and sperm, including the imprinting loci (Kobayashi et al., 2012) (Figure 4B). Most of these differences disappear during preimplantation period, while the gDMRs of the canonical imprinted regions are protected from such a global DNA demethylation wave (Figure 4C) (Kobayashi et al., 2012; Smallwood et al., 2011) and maintained in the postimplantation period (Figure 4D), indicating that the canonical genomic imprinted regions have been selected and conserved in mammals because of their evolutionary advantages. Interestingly, certain members of the Krüppel-associated box (KRAB)-containing zinc finger proteins (ZFPs), such as ZFP57 and ZNF445, play a key role in this process (Li et al., 2008; Takahashi et al., 2019). ZFP57 and ZNF445 bind a CpG-containing hexanucleotide motif present in multiple copies in most ICRs (Quenneville et al., 2011; Strogantsev et al., 2015; Anvar et al., 2016), and each forms a large complex that represses transcription by recruiting KRAB-associated protein1 [KAP1, aca tripartite motif containing 28 (TRIM28)], SET domain bifurcated histone lysine methyltransferase 1 (SETDB1), heterochromatin protein 1 (HP1) and DNMT1, and then maintain DNA methylation on the gDMRs of the newly replicated DNA by symmetric CpG methylation (Sharif et al., 2007; Quenneville et al., 2011; Liu et al., 2013; Mochizuki et al., 2021) (Figure 4C). The KRAB-ZFPs are evolutionarily diverse proteins that protect against the insertion of retroviruses (Macfarlan et al., 2012; Rowe et al., 2013), demonstrating that another host defense mechanism against retroviruses also exists and is functional in the maintenance of genomic imprinting memories (Edwards et al., 2019; Ondičova et al., 2020; Hanna and Kelsey, 2021). Thus, accumulating evidence indicates a close relationship between genomic imprinting and the insertion of retroviruses and LTR retrotransposons, as originally proposed in the host defense hypothesis. Importantly, newly invading DNAs are not completely repressed in both of the parental alleles because of the differential recognition mechanisms in the paternal and maternal germ cells, thereby leading to the complementary monoallelic expression of *PEGs* and *MEGs* (Figure 4D).

## Evolutionary Advantages of Genomic Imprinting

What is the evolutionary advantage of genomic imprinting? In other words, why have the canonical genomic imprinted regions been selected and widely conserved in therian mammals? We would like to address this issue based on the complementary monoallelic expression mechanism. First, this arrangement allows for the expression of all of the genes in the imprinted regions as *PEGs* and *MEGs* by controlling for the *cis*-elements in the gDMRs (Figure 1). As mentioned earlier, the *cis*-elements involved in the newly inserted gDMR sequences (Figure 3) exert a long-range effect on nearby genes, and thus repress a substantial number of resident genes. However, DNA methylation of the *cis*-elements in one of the alleles allows for recovery of the expression of such resident genes, so DNA methylation is required for the expression of certain imprinted genes (Figures 1, 2). As several of the *PEGs* and *MEGs* play an essential role in the current mammalian developmental system, their monoallelic expression would be expected to be disadvantageous in a general sense. However, under a special constraint, such as described for the *cis*-elements, monoallelic expression affords an advantage, because even a limited monoallelic expression is preferable to the complete loss of expression (Figures 1, 2). This indicates that genomic imprinting arose as a consequence of an evolutionary trade-off for survival (Kaneko-Ishino and Ishino 2015, 2019). This may also account for why the canonical imprinted regions have been so widely conserved in the therian mammals.

As mentioned in the introduction, it is likely that the conflict over maternal resources exerts pressure on *PEGs* promoting and *MEGs* repressing embryonic growth. In most cases, at least one imprinted gene in each canonical imprinted region seems to fit the conflict/kinship hypothesis (Moore and Haig, 1991; Wilkins and Haig 2003; Haig 2004) although most imprinted genes appear to be bystanders, that is, they are simply involved in this process as bystander genes (Barlow and Bartolomei 2014). In addition, it is also likely that gene dosage regulation of the evolutionarily conserved resident imprinted genes is another important factor in selection, because it must have contributed to the formation of the current mammalian developmental system by increasing pre-and postnatal fitness (Charalambous et al., 2012; Ball et al., 2013; Wolf et al., 2014).

Other hypotheses have been proposed that suggest genomic imprinting arose for the prohibition of parthenogenetic development or protection against the development of malignant placental tissue in such parthenogenetic embryos (Solter 1988; Varmuza and Mann 1994). Although it is difficult to prove these hypotheses by experiment, these features are surely advantageous for viviparous mammals and seem to have been acquired from the beginning via genomic imprinting, because the first two imprinted regions established in a common therian ancestor were the *PEG10* and *H19/IGF2* regions (Figure 3), that exert potent effects on placental formation and growth, respectively. This also supports the placenta hypothesis (Hall 1990; Kaneko-Ishino et al., 2003), which proposes that genomic imprinting is deeply related to the emergence of the placenta in the course of evolution, because a considerable number of imprinted genes play important roles in the placenta. In addition, H3K27me3 based non-canonical imprinting is unique to the placenta, and contributes to placental development and growth in a species-specific manner, such as *Xist, Gab1* and *Sfmbt2* in mice (Itoh et al., 2000; Okae et al., 2014; Inoue et al., 2017a, 2017b, 2018; Hanna et al., 2019; Inoue et al., 2020; Hanna and Kelsey 2021).

**FIGURE 5:**
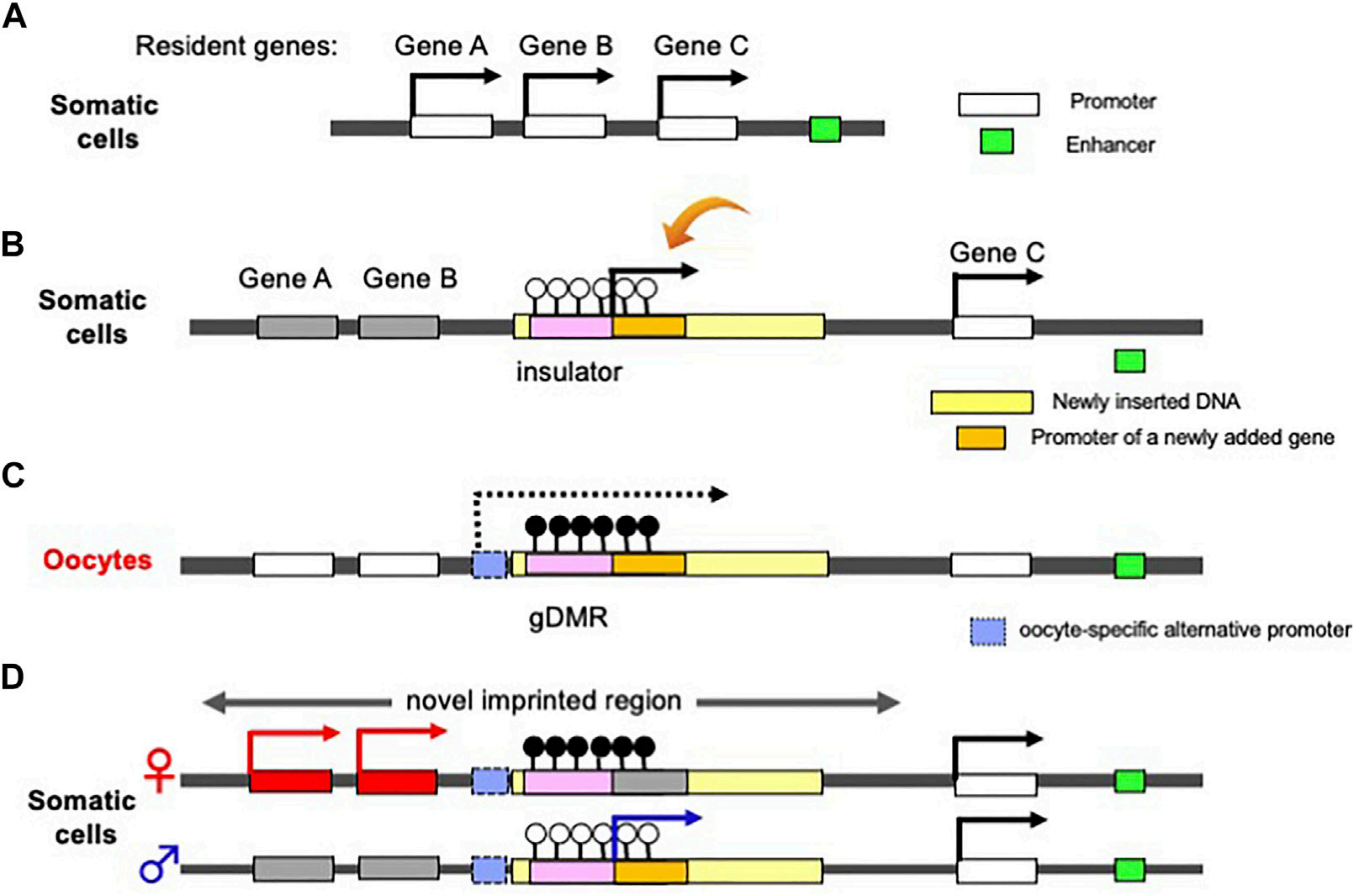
Generation of a novel canonical imprinted region. **(A)** Before an insertion event. A chromosomal region comprising three non-imprinted evolutionary resident Genes A, B and C. A downstream enhancer sequence (green) regulates the activation of the three genes. **(b)** Insertion of a large DNA fragment (yellow) containing an insulator sequence as a critical *cis*-element (pink) and a novel gene (orange) between Genes B and C. The insertion event leads to the repression of Genes A and B via the insulator function (see Figure 1). The novel added gene is expressed from the newly integrated DNA fragment in addition to Gene C. **(C)** DNA methylation on the inserted insulator in oocyte. Emergence of an upstream promotor (light blue) that expresses an oocyte-specific alternative transcript (dashed line). This transcript goes through the insulator, leading to DNA methylation on the insulator in an oocyte-specific manner. **(D)** Expression of a newly added gene from paternal allele of a novel imprinted region (arrow), while Genes A and B from maternal allele because the gDMR in the paternally-derived chromosome is not DNA methylated.

Finally, we would like to address another benefit of the complementary monoallelic mechanism. It provides a means of expression for both newly inserted genes as well as the conserved, already resident genes, therefore, allows for the acquisition of new genes in the canonical imprinted regions, and genes like *PEG10* and *H19* (Figure 5). Therefore, genomic imprinting may have made a contribution to the innovation of the emergent mammalian functions. Such newcomer genes may be separated into two groups: 1) newly acquired genes from retroviruses or of unknown origin, and 2) genes duplicated by retroposition. Among these genes, several play essential/important roles in development, growth and behavior, as discussed in the following two sections.

## Novel Functions Provided by Newly Acquired Imprinted Genes in Mammals

In addition to *PEG10* and *H19*, there are several imprinted regions that are accompanied by novel acquired genes. In the Delta like non-canonical Notch ligand 1 (*DLK1*)-Iodothyronine deiodinase 3 (*DI O 3*) region, a large insertion comprising *MEG3,* Retrotransposon GAG-like 1 (*RTL1*)*/PEG11, antiRTL1/antiPEG11,* and *MEG8/*RNA imprinted and accumulated in nucleus (*RIAN*) and *MEG9/*microRNA-containing gene (*MIRG*) occurred in association with the emergence of an intergenic (IG)*-DMR* between *DLK1 and MEG3* in eutherians (Kobayashi et al., 2000; Miyoshi et al., 2000; Schmidt et al., 2000; Wylie et al., 2000; Charlier et al., 2001; Edwards et al., 2008). Thus, they are eutherian-specific genes: *RTL1/PEG11* is a protein coding gene while the other maternally expressed non-coding RNAs include a cluster of siRNA and/or snoRNAs (Charlier et al., 2001; Cavaillé et al., 2002). In the *PEG3* region, the *PEG3*-DMR localizes on the *PEG3* promoter and regulates *PEG3* itself and neighboring paternally expressed Ubiquitin specific peptidase 29 (*USP29*), as well as maternally expressed Zinc finger imprinted 1 (*ZIM1*) and *ZIM3* (Kim et al., 2001, 2007). *PEG3* is also a eutherian-specific acquired gene. *PEG3* has quite unique zinc finger motif in terms of its amino acid sequence and its intervals, and no homologous C2H2-type of zinc finger protein is present in marsupials, monotremes or other vertebrates (Kuroiwa et al., 1996). The construction of the *PEG3* imprinted region is polymorphic among eutherian species, that is, there are certain differences that occur in a species-specific manner (Kim et al., 2007). *NEURONATIN* (*NNAT,* aka *PEG5*) (Kagitani et al., 1997; Kikyo et al., 1997) is inserted within an intron of the non-imprinted Bladder cancer associated protein (*BCAP*) and its promoter became the *NNAT/PEG5-*DMR (John et al., 2001). *NNAT/PEG5* is another eutherian-specific gene encoding a proteolipid of unknown origin, and there is a single imprinted gene in this locus (Evans et al., 2005) (Table 1).

Interestingly, *PEG10* and *RTL1/PEG11* are derived from retrovirus *GAG* and *POL* (Charlier et al., 2001; Ono et al., 2001). They exhibit a certain similarity to the sushi-ichi LTR retrotransposon, thus have come to be referred to as retrotransposon-GAG like genes. In any event, it is likely that the “gypsy” type of LTR retrotransposon to which the suchi-ichi retrotoransposon belong is derived from a retrovirus (Kim et al., 1994; Song et al., 1994). *PEG3* was probably generated by fusion of the same retroviral GAG and a C2H2-type of zinc finger protein generated by gene duplication (Campillos et al., 2006). *PEG10* is essential for the induction of placenta-specific cells, i.e., the trophoblast cells in the spongiotrophoblast and labyrinth layers, so its deficiency causes early embryonic lethality (Ono et al., 2006). It also plays an essential role in the maintenance of the fetal capillary network in the labyrinth layer: mutant mice with defective PEG10 protease exhibit perinatal lethality due to severe damage of the entire fetal capillary network (Shiura et al., 2021). *PEG10* is also associated with progression of various cancers, such as lung, breast, liver, and pancreatic cancer (Okabe et al., 2003; Li et al., 2006). Recently, accumulation of the PEG10 protein was reported in neurons differentiated from induced pluripotent stem cells (iPSs) from Angelman syndrome (AS) patients, suggesting an important role in the brain (Pandya et al., 2021).

*RTL1/PEG11* plays essential roles in the placenta, muscle and brain and is the major gene responsible for the Kagami-Ogata and Temple syndromes, two genomic imprinting diseases. It is located on human chromosome 14 (Kagami et al., 2008; Sekita et al., 2008, Kitazawa et al., 2017, 2020, 2021). Both overexpression and deficiency lead to abnormalities in the placenta as well as a variety of neuromuscular and/or psychiatric symptoms (Kitazawa et al., 2020, 2021). In the placenta, severe defects of the fetal capillaries can lead to late embryonic lethality or growth retardation (Sekita et al., 2008; Kitazawa et al., 2017). It is likely that damage to neonatal respiration-related tissues such as the intercostal, abdominal and diaphragm muscles lead to neonatal lethality (Kitazawa et al., 2020), presumably associated with abnormality of motor neurons in the descending corticonuclear tract that innervates the cranial nerves regulating the respiration-related muscles. *RTL1/PEG11* is also expressed in neurons in the corpus callosum, hippocampal and anterior cerebral commissures as well as limbic system, such as in the hippocampus and amygdala, suggesting that their malfunction is related to the psychiatric symptoms that manifest in these diseases (Kitazawa et al., 2021).

*PEG3* is also essential for placental functions as well as in the fetal and adult brain. Its defect causes fetal growth retardation, a problem in suckling milk in the pups, and abnormal maternal-care behaviors due to defects in the milk release process in females (Li et al., 1999) and thermoregulation (Broad et al., 2009), while biallelic expression of *Peg3* leads to subtle impacts in male and female mice (Bretz and Kim 2017). Certain mouse *Peg3* mutations also cause sex-biased outcomes (Kim et al., 2013; He et al., 2016). *PEG3* functions as a tumor suppressor in glioma in the brain (Kohda et al., 2001; Maegawa et al., 2004; Jiang et al., 2010). The loss of *NNAT/PEG5* is associated with decreased cerebellar folding in mice (Kikyo et al., 1997) and leads to postnatal growth restriction and adult obesity (Millership et al., 2018). It is reported that aggregates of the NNAT/PEG5 protein cause neuronal loss in Lafora disease, diabetes, and cancer (Joseph, 2014).

## New Functions Provided by Imprinted Genes Duplicated by Retropositioning

NECDIN (*NDN*)*,* MAGE Family Member L2 (*MAGEL2*) and Makorin Ring Finger Protein 3 (*MKRN3*) are located in the PWS/AS imprinted region and are retroposed genes generated from the cDNAs of their paralogs (Rapkins et al., 2006) (Table 1). The retropositioning events are sometimes associated with the generation of newly imprinted regions (Wood et al., 2007), such as the nucleosome assembly protein 1-like 5 (*NAP1L5*) and Inositol Polyphosphate-5-Phosphatase F (*INPP5F_V2*) regions in eutherians, and also the Malignant T-cell-amplified sequence 2 (*MCTS2*) and U2 small nuclear RNA auxiliary factor 1-related sequence 1 (*U2AF1-RS1*) regions in a lineage- and species-specific manner. Their own promoter regions became DMRs and interestingly, their original genes were localized in the X-chromosome (Wood et al., 2007). These genes were introduced into the eutherian genome as imprinted genes, presumably via surprising and complex processes that exploited the complementary monoallelic expression mechanism of genomic imprinting (Figure 5).

Among these genes, *NDN, MAGEL2,* and *MKRN3* play essential roles in the brain. The former two genes are responsible for certain symptoms of PWS including neonatal lethality, prenatal growth retardation and postnatal growth abnormalities, such as, obesity (Lee et al., 2005; Bischof et al., 2007), and the latter gene is responsible for precocious puberty (Abreu et al., 2013; Li et al., 2021). MKRN3-mediated ubiquitin signaling controls expression of Gonadotropin releasing hormone 1 (*GNRH1*) at both transcriptional and post-transcriptional levels (Li et al., 2021). *MAGEL2* mutations causes Schaaf-Yang syndrome, a rare neurodevelopmental disorder that shares multiple clinical features with the genetically related PWS (Schaaf et al., 2013).

## Conclusion

Accumulating evidence has painted a picture of a close relationship between genomic imprinting and invading DNA: 1) the insertion of DNA sequences corresponding to the gDMRs (Figure 3), 2) retroviral LTRs are used as promoters of oocyte-specific transcripts for DNA methylation on the gDMRs (Figure 4B), 3) the antiviral KRAB-ZFP system protects the gDMRs from global DNA demethylation (Figure 4C), providing strong support for the host defense hypothesis. Taken together, it is likely that each genomic imprinted region arose by chance as a consequence of an evolutionary trade-off for survival (Kaneko-Ishino and Ishino 2019) using the existing DNA methylation machinery against foreign DNA. Each region has been selected and conserved in therian/eutherian mammals for the advantage(s) it confers. We have also discussed another evolutionary advantage that is exerted by the complementary monoallelic expression mechanism which is that it affords a kind of genome innovation machinery that enables the introduction of novel genes via acquisition from retroviruses and also gene duplication by retroposition (Kaneko-Ishino and Ishino 2012; Edwards et al., 2019). As discussed in this review, in addition to the “Conflict” and “Complementation” activities, there are a variety factors acting in concert that make up the biological significance of genomic imprinting which remains a fascinating and critically important theme in mammalian biology.
